# The Cytochrome P450 Enzyme SsCyp64 Mediates γ-linolenyl Alcohol in Regulating Sexual Mating/Filamentation and Pathogenicity of *Sporisorium scitamineum*

**DOI:** 10.3390/jof11100729

**Published:** 2025-10-10

**Authors:** Enping Cai, Bo Xiong, Qiuping Ling, Xueting Li, Xinglong Chen, Changqing Chang, Jiayun Wu, Nannan Zhang

**Affiliations:** 1 Guangdong Sugarcane Genetic Improvement Engineering Center, Institute of Nanfan & Seed Industry, Guangdong Academy of Sciences, Guangzhou 510316, Chinachenxinglong567@126.com (X.C.); 2 Guangdong Provincial Key Laboratory of Microbial Signals and Disease Control, Engineering Research Center of Biological Control, Ministry of Education, College of Plant Protection, South China Agricultural University, Guangzhou 510642, China

**Keywords:** *Sporisorium scitamineum*, mating/filamentation, cytochrome P450 enzyme, γ-linolenyl alcohol

## Abstract

Sugarcane smut, caused by *Sporisorium scitamineum*, is a devastating fungal disease of sugarcane. Sexual mating/filamentation of opposite mating types is a key step in the infection and pathogenicity of *S. scitamineum*, yet its regulation remains unclear. In this study, we identified a cytochrome P450 enzyme-encoding gene, *SsCYP64*, which plays an important role in oxidative stress and maintaining cell membrane stability in *S. scitamineum*. Further investigations revealed that deletion of *SsCYP64* leads to a decrease in the transcriptional level of *SsPRF1*, a key transcription factor regulating the sexual mating of *S. scitamineum*. Subsequently, the constitutive expression of *SsPRF1* restored the defect in sexual mating/filamentation of the *SsCYP64* deletion mutant, indicating that SsCyp64 regulates the sexual reproduction of *S. scitamineum* by mediating the transcriptional level of *SsPRF1*. In addition, metabolomic analysis revealed that the fatty alcohol metabolite γ-linolenyl alcohol significantly decreased in the *SsCYP64* deletion mutant, whereas exogenous supplementation with γ-linolenyl alcohol increased the transcriptional level of *SsPRF1* and partially restored the sexual mating/filamentation of the *SsCYP64* deletion mutant. In conclusion, our results indicated that SsCyp64 mediated the transcription of *SsPRF1* by modulating γ-linolenyl alcohol levels, thereby regulating the formation of dikaryotic hyphae in *S. scitamineum*. These findings provide new insights into the role of cytochrome P450 enzymes in the pathogenic process of plant pathogenic fungi.

## 1. Introduction

Sugarcane (*Saccharum* spp.) is a globally critical crop that contributes to both sugar production and bioenergy generation [1]. However, it is highly susceptible to smut disease caused by the fungal pathogen *Sporisorium scitamineum*. This disease severely reduces yield and quality and poses a significant threat to the sustainable development of the sugar industry [2]. The sexual mating/filamentation of haploid cells with opposite mating types to form dikaryotic hyphae is a key step in the infection and pathogenicity of *S. scitamineum* [3,4]. Studies have shown that the deletion of the pheromone-responsive transcription factor *SsPRF1* leads to the complete loss of the ability to form dikaryotic hyphae and pathogenicity [5]. Furthermore, *SsPRF1* regulates the expression of *a*- and *b*-locus genes critical for dikaryotic hyphae formation [6]. In conclusion, the study indicates that *SsPRF1* plays an important role in regulating the sexual mating/filamentation and pathogenicity of *S. scitamineum*.

Cytochrome P450 (CYP) superfamily enzymes are widely distributed in organisms and play a key role in the synthesis of secondary metabolites and the metabolism of exogenous substances [7]. Among them, the Cyp64 subfamily, as a member of the CYP superfamily, has been identified in fungi and is involved mainly in the biosynthesis of mycotoxins and secondary metabolites [8]. In *Aspergillus parasiticus* and *Aspergillus flavus*, the *CYP64* homologous genes *ordA* or *aflQ* are core components of the aflatoxin biosynthesis gene cluster, and the cytochrome P450 monooxygenase encoded by these genes catalyzes the conversion of O-methylsterigmatocystin (OMST) to aflatoxins B_1_, G_1_, B_2_, and G_2_ [9,10,11,12]. This enzymatic reaction involves multiple steps such as oxidation, rearrangement, and demethylation, indicating the key role of Cyp64 in the aflatoxin biosynthesis stage. In *Thermomyces dupontii*, the fusion protein containing the Cyp64-like domain is involved in the biosynthesis of prenylated indole alkaloids, contributing to the formation of iron chelates and the regulation of iron metabolism [13], which indicates that it is crucial for fungi to adapt to changes in environmental temperature. In summary, Cyp64 enzymes are structurally and functionally conserved in fungi and mainly mediate oxidation reactions in secondary metabolic pathways, especially those that play a role in the biosynthesis of toxic metabolites and iron-chelating compounds, thereby affecting the pathogenicity and environmental adaptability of fungi. However, the mechanism of action of Cyp64 enzymes in smut fungi remains unclear.

Industrially, linolenyl alcohol (C_18_H_32_O) can be synthesized de novo using α-linolenic acid as a precursor by expressing fatty acyl-CoA reductase (MhFAR) in the engineered *Yarrowia lipolytica* [14], indicating that it is a polyunsaturated fatty alcohol derived from α-linolenic acid. In plant extracts, linolenyl alcohol has been identified as a major bioactive component. For example, linolenyl alcohol in the leaf extract of *Clinacanthus nutans* may have a high binding affinity for the p53-binding protein Mdm-2 [15], suggesting that it may play a role in regulating cancer cell proliferation. Antibacterial activity is another core function of linolenyl alcohol; it inhibits the growth of Gram-positive bacteria such as *Streptococcus mutans* by disrupting cell membrane integrity, but has little effect on Gram-negative bacteria [16]. In addition, linolenyl alcohol acts synergistically with aminoglycoside antibiotics to increase the bactericidal activity against *Streptococcus mutans* [17]. Linolenyl alcohol can inactivate *African swine* fever virus and inhibit its replication in Vero cells, indicated that it has antiviral properties [18]. However, the role of linolenyl alcohol in plant pathogenic fungi remains unclear.

In this study, we identified a cytochrome P450 enzyme-encoding gene *SsCYP64*, which plays an important role in oxidative stress, maintaining cell membrane stability, sexual reproduction, and pathogenicity in *S. scitamineum*. Further investigations revealed that SsCyp64 modulated the transcription of *SsPRF1* by mediating linolenyl alcohol levels, thereby regulating the sexual mating/filamentation of *S. scitamineum*. These findings provide new insights into the role of cytochrome P450 enzymes in the pathogenic process of plant pathogenic fungi.

## 2. Materials and Methods

### 2.1. Domain Prediction and Phylogenetic Analysis

The amino acid sequence of the SsCyp64 protein was obtained from the NCBI Protein Database (https://www.ncbi.nlm.nih.gov/protein/, accessed on 19 September 2024; accession number: CDS82158.1). The domain of SsCyp64 was predicted using the SmartBLAST tool (https://blast.ncbi.nlm.nih.gov/smartblast/, accessed on 19 September 2024), and the domain distribution profile was constructed using IBS 1.0.1 software. A phylogenetic analysis of Cyp64 proteins from selected basidiomycetous and ascomycetous species was carried out via the Maximum Parsimony method in MEGA 7. Bootstrap support values derived from 1000 resampling replicates are displayed on the respective branches.

### 2.2. Strains and Culture Conditions

The wild-type strains of the sugarcane smut fungus, *S. scitamineum* (*MAT-1* and *MAT-2*), which represent opposite mating types, were isolated and characterized by Yan and maintained in our laboratory [4]. Details of the strains used in this study are listed in Table S1. The culture conditions for the haploid strains were based on previously described YePS medium. The detailed protocol was as follows: the strains were activated on YePSA medium (Yeast extract–Peptone–Sucrose–Agar, pH 6.5) supplemented with Ampicillin (100 μg/mL) or Cefotaxime (100 μg/mL) and incubated at 28 °C for 24–48 h. A small amount of haploid sporidia was then harvested from the culture plate and transferred to 5 mL of sterile YePS medium, followed by shaking incubation at 28 °C and 200 rpm overnight to produce the seed culture.

### 2.3. Strain Construction and Identification

Deletion of *SsCYP64*: 1.5 kb DNA fragments corresponding to the upstream and downstream regions of *SsCYP64* were selected and designated as the left homologous arm (LB) and the right homologous arm (RB), respectively. The left and right homologous arms were amplified via the primer pairs *SsCYP64*-LB-F/*SsCYP64*-LB-R and *SsCYP64*-RB-F/*SsCYP64*-RB-R. Subsequently, the left homologous arm was fused with the truncated sequence *HYG^R^*-up of the hygromycin resistance gene (*HYG^R^*), whereas the right homologous arm was fused with *HYG^R^*-down. The resulting DNA constructs containing the truncated *HYG^R^* gene were introduced into the protoplasts of the wild-type *MAT-1* and *MAT-2* strains through PEG-mediated transformation, as described previously. The transformants were selected on YePSS (Yeast extract–Peptone–Sucrose–Sorbitol, pH 6.5) agar medium supplemented with 200 μg/mL Hygromycin B (Merck, St. Louis, MO, USA). Protoplast isolation and transformation procedures were carried out in accordance with the protocol described in reference [6]. After three rounds of resistance screening, the obtained transformants were analyzed by PCR using the primers *SsCYP64*-in-F/*SsCYP64*-in-R and *SsCYP64*-out-F/*SsCYP64*-out-R to confirm correct genomic integration. Additionally, reverse transcription quantitative PCR (RT-qPCR) was conducted using primers RT-qPCR-*SsCYP64*-F/RT-qPCR-*SsCYP64*-R to evaluate the efficiency of gene deletion. All primers used in this study are summarized in Table S2.

Complementation of *SsCYP64*: PCR amplification was performed via the primer pair *SsCYP64*-com-F/*SsCYP64*-com-R with genomic DNA from the wild type as the template. The amplified fragment encompassed a 1.4 kb open reading frame (ORF) of *SsCYP64* as well as the complete *SsCYP64*. The PCR product was subsequently cloned and inserted into the pEASY-COM vector via the ClonExpress II One Step Cloning Kit (Vazyme, Nanjing, China), yielding the recombinant plasmid pEASY-COM-*SsCYP64*. Using this plasmid as the template, two DNA fragments, COM-*SsCYP64*-LB and COM-*SsCYP64*-RB, containing truncated sequences of the Zeocin resistance (*ZEO^R^*) marker were amplified using primer pairs COM-LB-F/COM-LB-R and COM-RB-F/COM-RB-R [19], respectively. These fragments were subsequently cotransformed into protoplasts of *sscyp64*Δ*-1* and *sscyp64*Δ*-2* via PEG-mediated transformation. The transformants were selected on medium supplemented with 100 μg/mL Zeocin (Invitrogen, Carlsbad, CA, USA). The resulting complemented strains, designated *sscyp64*Δ/*CYP64-1* and *sscyp64*Δ/*CYP64-2*, were validated by PCR amplification using the primers *SsCYP64*-in-F/*SsCYP64*-in-R. Finally, RT-qPCR was conducted using the primers RT-qPCR-*SsCYP64*-F/RT-qPCR-*SsCYP64*-R to confirm the re-expression of *SsCYP64*. All sequences of primer used in this study are summarized in Table S2.

Constitutive expression of *SsPRF1* in the *SsCYP64* deletion mutant: PCR amplification was carried out using the primer pair *SsPRF1*-con-F/*SsPRF1*-con-R with cDNA as the template. The amplified fragment encompassed the full-length coding sequence of *SsPRF1*. The PCR product was subsequently cloned and inserted into the pEASY-OE vector via the ClonExpress II One Step Cloning Kit (Vazyme, Nanjing, China) following the protocol described in reference [19], yielding the recombinant plasmid pEASY-OE-*SsPRF1*. Using this plasmid as the template, two DNA fragments, con-*SsPRF1*-LB and con-*SsPRF1*-RB, containing truncated sequences of the *ZEO^R^* marker were amplified via the primer pairs con-LB-F/con-LB-R and con-RB-F/con-RB-R, respectively. These fragments were subsequently cotransformed into protoplasts of *sscyp64*Δ*-1* and *sscyp64*Δ*-2* via PEG-mediated transformation. The transformants were selected on a medium supplemented with 100 μg/mL Zeocin (Invitrogen, Carlsbad, CA, USA). The resulting transformants, designated *sscyp64*Δ/*con-PRF1-1* and *sscyp64*Δ/*con-PRF1-2*, were validated by RT-qPCR using the primers RT-qPCR-*SsPRF1*-F/RT-qPCR-*SsPRF1*-R to confirm the re-expression of *SsPRF1*. All sequences of primers used in this study are summarized in Table S2.

### 2.4. Extraction of Total DNA and RNA

The genomic DNA of *S. scitamineum* was extracted using the SDS-based method according to the protocol described [20]. Fresh haploid sporidia of *S. scitamineum* were cultured in YePS agar medium at 28 °C for 24 h, after which the cells were harvested for total DNA extraction. For total RNA extraction, fresh haploid sporidia of *S. scitamineum* were cultured in YePS medium and shaken at 28 °C and 200 rpm overnight to produce the seed culture. In accordance with the experimental requirements, the seeds were spread on different media and cultured at 28 °C until the required time. Then, the cells were collected, quickly frozen with liquid nitrogen, and ground into powder. Total RNA was extracted via the RNeasy Mini Kit (QIAGEN, Germantown, MD, USA), which strictly followed the manufacturer’s instructions to ensure high-quality RNA preparation.

### 2.5. RT-qPCR Analysis

RT-qPCR primers were designed using the PrimerQuest™ tool (https://sg.idtdna.com/pages/tools/primerquest?returnurl=%2Fprimerquest%2FHome%2FIndex, accessed on 16 September 2022). The GC content of the primers was set within the range of 45% to 55%, and the annealing temperature (Tm) was targeted between 55 °C and 65 °C. Amplification efficiency was assessed via the standard curve method, and only primers with efficiencies ranging from 90% to 110% were selected for subsequent experiments. Total RNA was reverse-transcribed into cDNA via the HiScript^®^ II First Strand cDNA Synthesis Kit (Vazyme, Nanjing, China). RT-qPCR was then carried out using a Roche qPCR system and ChamQ SYBR qPCR Master Mix (Vazyme, Nanjing, China), together with gene-specific primers. Amplification specificity was verified by melting curve analysis, which revealed a single peak with a Tm value exceeding 80 °C. Relative gene expression levels were calculated via the 2^−ΔΔ^*^CT^* method [21], with the cytoskeletal gene *ACTIN* used as the internal reference [19]. To ensure the reliability and reproducibility of the results, the experiment was conducted with three independent biological replicates, each containing two technical replicates.

### 2.6. Analysis of H_2_O_2_ and SDS Tolerance

A preprepared single-spore suspension of fresh haploid sporidia of *S. scitamineum* was inoculated into 0.5 mL of YePS medium and incubated at 28 °C with shaking at 200 rpm overnight. The culture mixture was then transferred to 2.0 mL of fresh YePS liquid medium and further incubated under identical conditions for 24 h. Following centrifugation at 4000 rpm for 5 min, the resulting cell pellet was resuspended in sterile distilled water and adjusted to an OD_600_ of 1.0. Serial 10-fold dilutions were subsequently performed, and 1.5 μL of each dilution was spotted onto two types of solid media: (1) YePSA medium (control group); and (2) YePSA medium supplemented with 1.8 mM H_2_O_2_ or 0.011% SDS (experimental group). After incubation at 28 °C for 3–4 days, differences in tolerance to H_2_O_2_ and SDS were evaluated by comparing colony growth phenotypes, and representative images were captured via a digital camera. The experiment was repeated three times as independent biological replicates, each with two technical replicates, to ensure the reliability and reproducibility of the results.

### 2.7. Mating/Filamentous Growth Assay

The haploid sporidia of *S. scitamineum* were inoculated into 0.5 mL of YePS liquid medium and incubated at 28 °C with shaking at 200 rpm overnight. The cultures were subsequently transferred to 2.0 mL of fresh YePS medium and further incubated under the same conditions for 24 h to reach the mid-logarithmic growth phase. Following incubation, the cells were harvested via centrifugation at 4000 rpm for 5 min and resuspended in sterile distilled water, after which the OD_600_ was adjusted to 1.0. Equal volumes of the two mating-type suspensions were mixed to obtain a 1:1 ratio. A 1.5 μL aliquot of the mixture was then spotted onto two types of solid media: YePSA medium, and (2) MM solid medium supplemented with or without γ-Linolenyl alcohol. The plates were incubated at 28 °C for 12–48 h, after which the formation of dikaryotic hyphae was assessed as an indicator of sexual mating/filamentation. Morphological observations were recorded using a Zeiss stereomicroscope or digital camera. The experiment was independently repeated three times as biological replicates, with two technical replicates per biological replicate.

### 2.8. Transcriptome Analysis

Mycelial samples of both the *SsCYP64* deletion mutant and the wild-type strain were collected after 0, 24, 48, and 72 h of induction in YePSA medium. These samples were subjected to total RNA extraction and subsequent transcriptomic analysis. mRNA was enriched from total RNA using Oligo dT magnetic beads, fragmented via fragmentation buffer, and reverse-transcribed into first-strand cDNA with random hexamer primers. Library preparation was carried out via the NEBNext^®^ Ultra™ RNA Library Prep Kit for Illumina^®^ platforms. De novo transcriptome assembly was performed using StringTie software v2.2.3, and the resulting novel transcript sequences were annotated by comparison with functional databases, including Pfam, SUPERFAMILY, Gene Ontology (GO), and KEGG. The assembled transcript fragments were mapped to the published reference genome of *S. scitamineum* to determine their genomic locations [22]. Differential gene expression analysis and functional annotation were conducted on the basis of alignment results against the NCBI Nonredundant (NR) and KOG databases.

### 2.9. Metabolomic Analysis

Mycelial samples of the *SsCYP64* deletion mutant and wild-type strains were collected after 0, 24, 48, and 72 h of sexual mating/filamentation in YePSA medium. A 100 mg aliquot of each sample was rapidly frozen in liquid nitrogen, ground into a fine powder, and transferred to an EP tube. Subsequently, 500 μL of 80% aqueous methanol was added, followed by vortex mixing, incubation on ice for 5 min, and centrifugation at 15,000× *g* for 20 min at 4 °C. The supernatant was diluted with mass spectrometry-grade water to achieve a final methanol concentration of 53%. Metabolomic profiling was conducted (Novogene Bioinformatics Technology Co., Ltd., Beijing, China) via ultrahigh-performance liquid chromatography coupled with high-resolution mass spectrometry [23,24]. The identified metabolites were annotated using the KEGG database (https://www.genome.jp/kegg/pathway.html, accessed on 10 April 2025), HMDB (https://hmdb.ca/metabolites, accessed on 10 April 2025), and LIPIDMaps database (http://www.lipidmaps.org/, accessed on 10 April 2025). Data processing was performed with the metabolomics analysis software metaX [25], followed by multivariate statistical analysis including principal component analysis (PCA) and partial least squares discriminant analysis (PLS-DA) to determine variable importance in projection (VIP) values for each metabolite [26,27]. Statistical significance between groups was assessed using *t*-tests (*p* values), and the fold changes (FC) in metabolite levels were calculated. Differentially abundant metabolites were selected on the basis of the following criteria: VIP > 1, *p* < 0.05, and FC ≥ 2 or FC ≤ 0.5. Volcano plots and bubble plots were generated via the R package ggplot2.

### 2.10. Pathogenicity Analysis

The haploid sporidia of *S. scitamineum* were inoculated into 0.5 mL of YePS liquid medium and incubated at 28 °C with shaking at 200 rpm for overnight. The cultures were then transferred to 5.0 mL of fresh YePS liquid medium and further incubated under the same conditions for 24 h to reach the mid-logarithmic growth phase. Following incubation, the cells were harvested by centrifugation at 4000 rpm for 5 min and resuspended in sterile distilled water, after which the OD_600_ was adjusted to 1.0. Equal volumes of the two mating-type cultures were mixed to obtain a 1:1 ratio. A 1.0 mL syringe was used to inject the mixed suspension into seedlings of the susceptible sugarcane variety XTT22, with each seedling receiving 200 μL of the inoculum. The experiment was independently repeated three times as biological replicates, with no fewer than 12 sugarcane seedlings per replicate.

### 2.11. Statistical Analysis

Statistical significance was assessed via one-way analysis of variance (ANOVA), followed by Bonferroni’s multiple comparison test to compare means. The results were considered statistically significant if the *p* value was <0.05. Histograms were generated using GraphPad Prism 7 software.

## 3. Results

### 3.1. SsCyp64 Positively Regulates Oxidative Stress in S. scitamineum, but Negatively Affects the Stability of the Cell Membrane

**Figure 1:**
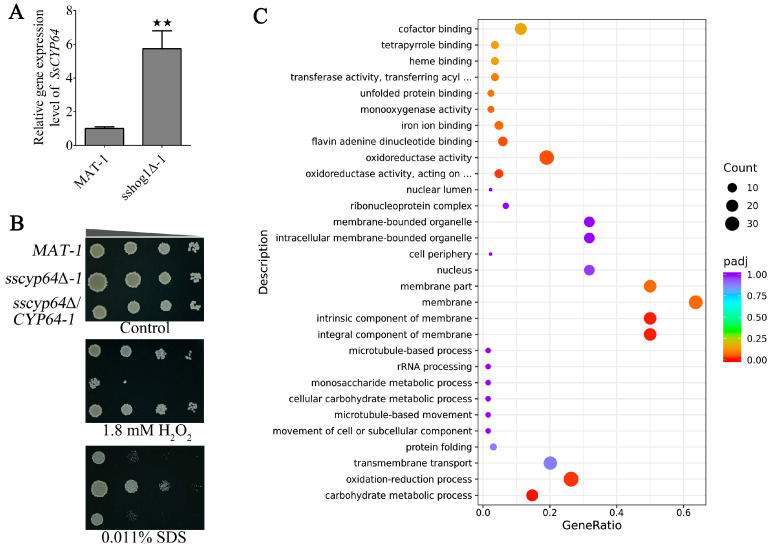
The role of SsCyp64 in oxidative stress response and cell membrane stability of *S. scitamineum*. (**A**) RT-qPCR was conducted to quantify the transcriptional levels of *SsCYP64* in *MAT-1* and *sshog1*Δ*-1*. Total RNA was isolated from *MAT-1* and *sshog1*Δ*-1* following 48 h of cultivation on YePSA medium. The analysis included three biological replicates, each consisting of three technical replicates. The error bars represent the means ± standard errors of the means (SEMs). Statistical significance was assessed via one-way analysis of variance (ANOVA) followed by Bonferroni’s multiple-comparison test (^★★^  *p* < 0.01; NS, not significant). (**B**) The tolerances of *MAT-1*, *sscyp64*Δ*-1*, and *sscyp64*Δ/*CYP64-1* to 1.8 mM H_2_O_2_ or 0.011% SDS were evaluated. The haploid cells of the indicated strains were allowed to grow untill an OD_600_ of 1.0 was reached, after which 10-fold diluted cells were subsequently spotted onto YePSA medium with or without H_2_O_2_ or SDS. Images were taken 3–4 days after cultivation. The triangles indicate 10-fold serial dilutions from left to right. (**C**) Gene Ontology (GO) functional enrichment analysis of the transcriptome in the *sscyp64*Δ*-1* vs. *MAT-1* comparison. The haploid cells of the *sscyp64*Δ*-1* and *MAT-1* strains were allowed to grow to an OD_600_ of 1.0, and then spread on YePSA medium and cultured for 24 h. The fungal cells were subsequently collected for transcriptome sequencing. The size of each circle corresponds to the extent of enrichment, and the color gradient reflects the statistical significance. A *p*-value < 0.05 was considered statistically significant.

Our previous study demonstrated that the MAP kinase Hog1 participates in the oxidative tolerance mechanism of *S. scitamineum* by regulating the cytochrome P450 pathway [28]. We further found that deletion of *SsHOG1* increases the transcriptional level of *CDU26122.1* (Figure 1A) under conditions without any stress treatment. Subsequent NCBI BLASTp analysis of the protein sequence encoded by *CDU26122.1* confirmed its classification within the cytochrome P450 enzyme family 64, leading to its designation as *SsCYP64*. Phylogenetic analysis further revealed that SsCyp64 and its homologs from various basidiomycetous and ascomycetous species are highly conserved during evolution, particularly within the Ustilaginomycetes (smut fungi) clade, whereas they exhibit a more distant evolutionary relationship with homologs from ascomycetous species (Figure S1A). Domain analysis revealed that SsCyp64 contains a low-complexity region (LCR) and two evolutionarily conserved P450 domains (Figure S1B).

To further elucidate the functional significance of SsCyp64 in *S. scitamineum*, we generated targeted *SsCYP64* deletion mutants (*sscyp64*Δ*-1* and *sscyp64*Δ*-2*) along with corresponding genetic complementation strains (*sscyp64*Δ/*CYP64-1* and *sscyp64*Δ/*CYP64-2*) through homologous recombination. The successful deletion and reintroduction of the *SsCYP64* gene were validated by PCR and RT-qPCR analyses (Figure S2), with detailed strain information provided in Table S1. We subsequently evaluated the role of SsCyp64 in oxidative stress resistance and cell membrane stability. Compared with the wild-type *MAT-1* strain and the complemented strain *sscyp64*Δ/*CYP64-1*, the *sscyp64*Δ*-1* mutant exhibited significantly impaired growth on YePSA medium supplemented with 1.8 mM hydrogen peroxide (H_2_O_2_) while demonstrating enhanced tolerance to 0.011% SDS, a detergent known to compromise membrane integrity (Figure 1B). Transcriptomic analysis comparing *sscyp64*Δ*-1* and *MAT-1* revealed significant enrichment of differentially expressed genes (DEGs) in functional categories such as redox processes, oxidoreductase activity, and membrane-associated cellular components (Figure 1C).

### 3.2. SsCyp64 Regulates the Mating/Filamentation of S. scitamineum by Mediating SsPRF1 Transcription

**Figure 2:**
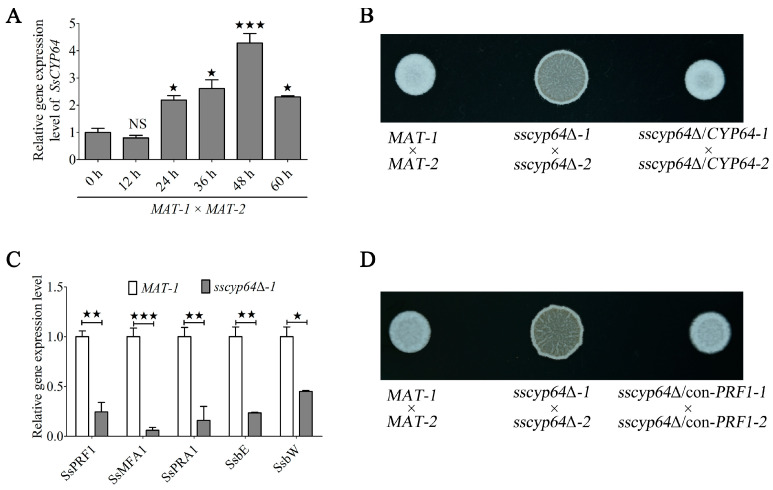
The role of SsCyp64 in the mating/filamentation and *SsPRF1* transcription of *S. scitamineum*. (**A**) RT-qPCR was conducted to analyze the transcriptional profile of *SsCYP64* during mating/filamentation in *S. scitamineum*. Total RNA was isolated from wild-type *MAT-1* × *MAT-2* at 0, 12, 24, 36, 48, and 60 h during mating/filamentation, and RT-qPCR was performed with *ACTIN* as an internal reference gene. The experiment included three biological replicates, each with three technical replicates. The error bars represent the means ± standard errors of the means (SEMs). Statistical significance was assessed via one-way analysis of variance (ANOVA) followed by Bonferroni’s multiple-comparison test (^★^ *p* < 0.05; ^★★★^ *p* < 0.001; NS, not significant). (**B**) Mating assay of the wild-type, *SsCYP64* deletion mutant, and complementation strains. The haploid cells of the indicated strains were mixed (*MAT-1* × *MAT-2*, *sscyp64*Δ*-1* × *sscyp64*Δ*-2*, and *sscyp64*Δ*/CYP64-1* × *sscyp6464*Δ*/CYP64-1*) and spotted onto YePSA plates. Following 48 h of incubation at 28 °C, colony morphology was recorded via a camera. (**C**) RT-qPCR analysis of *SsPRF1* and its downstream target genes in the *MAT-1* and the *sscyp64*Δ*-1* strains. The haploid cells of the *sscyp64*Δ*-1* and *MAT-1* strains were allowed to grow to an OD_600_ of 1.0, and then spread on YePSA medium and cultured for 48 h. The fungal cells were subsequently collected for RNA extraction and RT-qPCR analysis. *ACTIN* was used as the internal control. Three independent biological replicates were performed, each consisting of three technical replicates. The error bars indicate the means ± standard errors of the means (SEMs). Statistical differences were evaluated using one-way ANOVA with Bonferroni’s multiple-comparison test (^★^ *p* < 0.05; ^★★^ *p* < 0.01; ^★★★^ *p* < 0.001). (**D**) Mating assay of the wild-type, *SsCYP64* deletion mutant, and constitutively expressed *SsPRF1* strains. The haploid cells of the indicated strains were mixed (*MAT-1* × *MAT-2*, *sscyp64*Δ*-1* × *sscyp64*Δ*-2*, and *sscyp64*Δ/*con-PRF1-1* × *sscyp64*Δ/*con-PRF1-2*) and spotted onto YePSA plates. Following 48 h of incubation at 28 °C, colony morphology was recorded via a camera.

The formation of dikaryotic hyphae through sexual mating/filamentation represents a crucial developmental process for the pathogenicity of *S. scitamineum* [4]. To explore the potential role of *SsCYP64* in this biological event, we analyzed its transcriptional profile during dikaryotic hyphae formation. Our results demonstrated that transcription of *SsCYP64* was markedly upregulated during this process, peaking at 48 h post-induction (Figure 2A). We further evaluated the effect of *SsCYP64* deficiency on the production of dikaryotic hyphae on YePSA medium. Consistent with our expectations, spore mixtures of *sscyp64*Δ*-1* and *sscyp64*Δ*-2* produced only minimal dikaryotic hyphae at 48 h, a white colony phenotype significantly observed in the wild-type *MAT-1* × *MAT-2* or in the complemented strains *sscyp64*Δ/*CYP64-1* × *sscyp64*Δ/*CYP64-2* (Figure 2B). To elucidate the molecular mechanism underlying SsCyp64-mediated regulation of dikaryotic hyphae formation, we examined the expression levels of key regulatory and mating-type genes involved in this developmental pathway. Notably, the pheromone response transcription factor gene *SsPRF1*, along with its downstream mating-type genes at the *a*-(*SsMFA1* and *SsPRA1*) and *b*-(*SsbE* and *SsbW*) loci, presented significantly reduced transcription levels (Figure 2C). These results suggested that the defect in hyphal formation observed in *SsCYP64* deletion mutants may stem from the diminished transcription of *SsPRF1*. To verify this hypothesis, we constitutively expressed *SsPRF1* in the *SsCYP64* deletion background and established two independent complementation strains (*sscyp64*Δ/*con-PRF1-1* and *sscyp64*Δ/*con-PRF1-2*) (Figure S3). Notably, the restoration of *SsPRF1* expression largely rescued the defect in dikaryotic hyphae formation (Figure 2D).

### 3.3. SsCyp64 Mediates γ-Linolenyl Alcohol Synthesis to Regulate the Formation of Dikaryotic Hyphae in S. scitamineum

**Figure 3:**
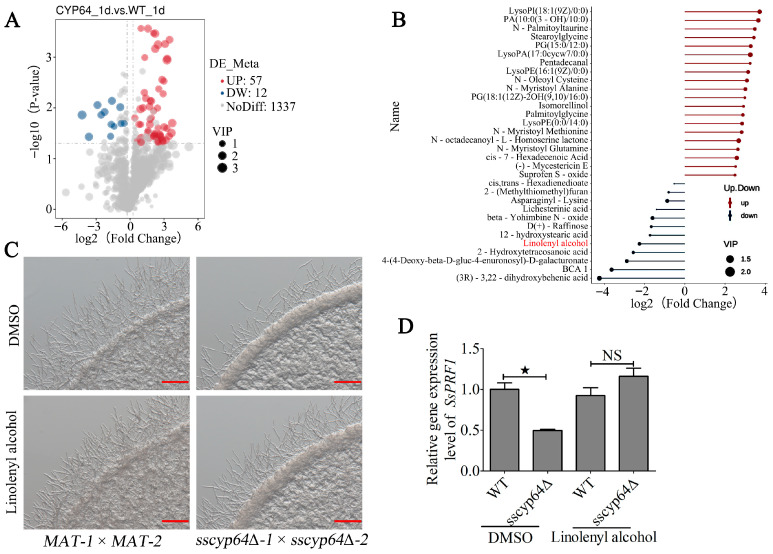
The role of γ-Linolenyl alcohol in the mating/filamentation and *SsPRF1* transcription of *SsCYP64* deletion mutant. (**A**) The overall distribution of differentially abundant metabolites in the CYP64-1 d vs. WT-1 d comparison group was visualized via a volcano plot. The *x*-axis represents the log2-fold change in each metabolite in the comparison group, whereas the *y*-axis indicates the negative log10-transformed *p*-value (*p*-value < 0.05). The size of each dot corresponds to the VIP (variable importance in projection) value derived from the OPLS-DA model, with larger dots indicating higher VIP values. The color of the dots reflects the direction of regulation: red for upregulated, blue for downregulated, and gray for nonsignificant metabolites. (**B**) Statistical analyses were performed on differentially abundant metabolites in the CYP64-1 d vs. WT-1 d comparison group which were visualized via a fireplot. The top 20 metabolites with the highest upregulation levels and all downregulated metabolites were selected for visualization. Blue represents downregulation, and red represents upregulation. The *x*-axis displays the log2-transformed fold change, and the color intensity of each dot represents the corresponding VIP value. The thresholds were set as VIP > 1.0, FC > 1.2 or FC < 0.833, and *p*-value < 0.05. (**C**) Evaluation of the effects of exogenous supplementation with γ-linolenyl alcohol on mating/filamentation in wild-type and *SsCYP64* deletion mutants. The haploid cells of the indicated strains were mixed (*MAT-1* × *MAT-2* and *sscyp64*Δ*-1* × *sscyp64*Δ*-2*) and spotted onto minimal medium supplemented with DMSO or 10.0 μM γ-linolenyl alcohol. Following 18 h of incubation at 28 °C, colony morphology was captured via a Zeiss stereomicroscope with a scale bar of 1.0 mm. (**D**) RT-qPCR analysis of *SsPRF1* transcriptional levels following exogenous supplementation with γ-linolenyl alcohol. The haploid cells of the indicated strains were mixed (*MAT-1* × *MAT-2* and *sscyp64*Δ*-1* × *sscyp64*Δ*-2*) and spread onto minimal medium supplemented with either DMSO or γ-linolenyl alcohol. After 36 h of incubation at 28 °C, total RNA was extracted and RT-qPCR was performed with *ACTIN* as an internal reference gene. Three independent biological replicates were conducted, each with three technical replicates. The error bars represent the means ± standard errors of the means (SEMs). Statistical significance was assessed via one-way ANOVA followed by Bonferroni’s multiple-comparison test to compare group means (^★^ *p* < 0.05; NS, not significant).

To further investigate the functional relationship between *SsCYP64* and *SsPRF1*, we performed a metabolomic analysis on the wild-type (*MAT-1* × *MAT-2*) and *SsCYP64* deletion mutant (*sscyp64*Δ*-1* × *sscyp64*Δ*-2*). The results revealed 57 upregulated and 12 downregulated differentially abundant metabolites in the *SsCYP64* deletion mutant compared with the wild type (Figure 3A). Visualization analysis of these metabolites revealed that 32 of them exceeded the average abundance across all measured variables (Figure 3B). To assess the effects of the downregulated metabolites on sexual mating/filamentation, we exogenously supplemented these metabolites (γ-linolenyl alcohol, 2-(Methylthiomethyl)furan, D(+)-Raffinose, and 12-hydroxystearic acid) in the *SsCYP64* deletion mutant. Notably, the addition of γ-linolenyl alcohol significantly increased the degree of sexual mating/filamentation of the *SsCYP64* deletion mutant to form dikaryotic hyphae (Figure 3C), whereas the other tested metabolites had no observable effect on sexual mating/filamentation (Figure S4), suggesting that SsCyp64 regulates sexual mating/filamentation by modulating γ-linolenyl alcohol levels. To elucidate the molecular mechanism underlying the effect of γ-Linolenyl alcohol on dikaryotic hyphae formation, we examined the transcriptional response of *SsPRF1* upon exogenous application of γ-linolenyl alcohol in the wild-type (*MAT-1* × *MAT-2*) and *SsCYP64* deletion mutant (*sscyp64*Δ*-1* × *sscyp64*Δ*-2*) backgrounds. The results demonstrated that γ-linolenyl alcohol supplementation restored the transcription levels of *SsPRF1* in the *SsCYP64* deletion mutant (Figure 3D).

### 3.4. SsCyp64 Is Essential for the Pathogenicity of S. scitamineum

**Figure 4:**
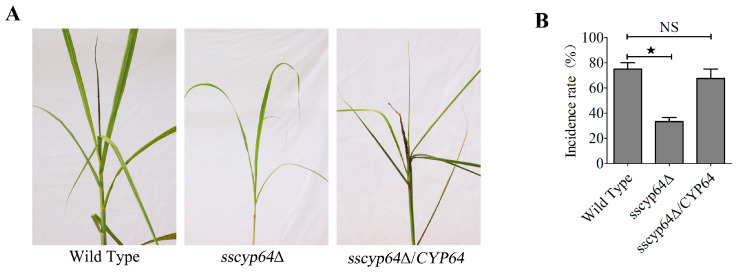
SsCyp64 plays a crucial role in the pathogenicity of *S. scitamineum*. (**A**) Pathogenicity assay of the wild-type, *SsCYP64* deletion mutant, and complementation strains. The symptoms of ‘black whip’ were documented and photographed at 3–6 months post-inoculation. The haploid cells of the indicated strains were mixed (*MAT-1* × *MAT-2*, *sscyp64*Δ*-1* × *sscyp64*Δ*-2*, and *sscyp64*Δ/*CYP64-1* × *sscyp6464*Δ/*CYP64-1*) in equal volumes and injected into the vicinity of the stem growing points of 5-leaf-stage seedlings of the susceptible sugarcane variety XTT22. (**B**) Incidence rates of sugarcane after infection with the wild-type, *SsCYP64* deletion mutant, and complementation strains. Disease incidence was calculated as (number of infected sugarcane plants/total number of plants) * 100%. The experiment consisted of three biological replicates, each comprising 12 technical replicates. The error bars represent the means ± standard errors of the means (SEMs). The statistical significance of differences was evaluated via one-way analysis of variance (ANOVA) followed by Bonferroni’s multiple-comparison test ( ^★^ *p* < 0.05; NS, not significant).

Given that SsCyp64 is involved in regulating the formation of dikaryotic hyphae in *S. scitamineum*, we investigated its potential role in pathogenic development. Five-leaf-stage seedlings of the susceptible sugarcane variety XTT22 were inoculated with the wild-type (*MAT-1* × *MAT-2*), *SsCYP64* deletion mutant (*sscyp64*Δ*-1* × *sscyp64*Δ*-2*), or *SsCYP64* complemented strain (*sscyp64*Δ/*CYP64-1* × *sscyp64*Δ/*CYP64-2*). The results revealed that both the wild-type and complemented strains induced typical “black whip” symptoms in sugarcane seedlings (Figure 4A), accompanied by a high incidence rate (Figure 4B). In contrast, the *SsCYP64* deletion mutants presented a significantly reduced disease incidence of only approximately 30% (Figure 4B). The complemented strains restored the disease incidence to approximately 70%, which was not significantly different from that of the wild-type strain (Figure 4A,B).

## 4. Discussion

This study identified a novel regulatory pathway, in which the cytochrome P450 enzyme SsCyp64 mediates γ-linolenyl alcohol levels to modulate the transcription of the pheromone response factor *SsPRF1*, thereby regulating the sexual mating/filamentation and pathogenicity of *S. scitamineum*. These findings expand our understanding of the functional diversity of cytochrome P450 enzymes in fungal pathogenesis and reveal metabolic signals as key drivers of developmental transitions in plant pathogenic fungi.

Cytochrome P450 enzymes of the CYP64 subfamily are conserved across fungi and play well-characterized roles in secondary metabolism, particularly in mycotoxin biosynthesis [8]. In *T. dupontii*, a Cyp64-like domain contributes to prenylated indole alkaloid synthesis, regulating iron metabolism and environmental adaptation [13]. Our study extends this functional repertoire by demonstrating that SsCyp64 integrates metabolic regulation (γ-linolenyl alcohol synthesis) with developmental signaling (*SsPRF1*-mediated mating/filamentation). This differs from its counterparts in ascomycetes, which primarily affect toxin production: in *A. parasiticus* and *A. flavus*, CYP64 homologs (OrdA/AflQ) catalyze critical steps in aflatoxin biosynthesis [10,11]. Furthermore, phylogenetic analysis revealed that SsCyp64 clusters closely with homologs from smut fungi, whereas it has a distant evolutionary relationship with homologs from ascomycete species, suggesting evolutionary specialization within this clade. This divergence may reflect adaptive evolution, as smut fungi rely on precise control of dikaryotic hyphae formation for biotrophic infection. This is supported by our results demonstrating that SsCyp64 is essential for sexual mating/filamentation and pathogenicity of *S. scitamineum*.

γ-Linolenyl alcohol is a polyunsaturated fatty alcohol derived from α-linolenic acid [14]. It exhibits anti-cancer activity in plant extracts [15], functions in antibacterial defense in bacteria [16], and participates in pheromone biosynthesis in insects [29], respectively. In this study, our findings provide new evidence for the regulation of fungal pathogenicity by fatty acid-derived signals. This is similar to how linolenic acid regulates sexual mating/filamentation in *S. scitamineum* [19], and how lipid droplets function as key determinants of pathogenicity in *Magnaporthe oryzae* [30,31]. These results demonstrated that γ-linolenyl alcohol plays an unprecedented role in linking lipid metabolism and developmental signaling. Notably, we found that the transcription of the pheromone-responsive transcription factor SsPrf1 is regulated by γ-linolenyl alcohol. This expands the known regulatory network of *SsPRF1*, which was previously thought to respond primarily to pheromone signaling via the MAPK and cAMP/PKA pathways [6,32]. In addition, linolenyl alcohol is a key precursor for the biosynthesis of sex pheromones in *Hyphantria cunea* [29]. Therefore, whether γ-linolenyl alcohol acts as a precursor for pheromone synthesis in *S. scitamineum* or directly regulates sexual mating is a question worthy of future exploration. Potential mechanisms include interaction with a transcriptional coactivator, modulation of chromatin accessibility, or activation of upstream signaling kinases. Furthermore, the integration of metabolic signals (γ-linolenyl alcohol) with canonical pheromone signaling likely enhances fungal adaptability during infection. For example, host-derived fatty acids may be metabolized into γ-linolenyl alcohol, which in turn triggers the activation of *SsPRF1* and filamentation. This hypothesis is supported by findings that metabolic sensors coordinate appressorium formation with nutrient availability in *M. oryzae* [33]. Once it is confirmed that *S. scitamineum* utilizes γ-linolenyl alcohol derived from sugarcane to promote infection, this compound could serve as a biomarker for future sugarcane disease-resistant breeding.

In addition to its role in sexual reproduction, SsCyp64 contributes to oxidative stress resistance and negatively regulates cell membrane stability. *SsCYP64* deletion mutants exhibit hypersensitivity to H_2_O_2_ but enhanced tolerance to SDS, a phenotype mirrored by transcriptomic enrichment of redox and membrane-associated genes. These findings align with the broad functions of cytochrome P450 enzymes in detoxification and lipid metabolism [34,35]. The dual roles of SsCyp64, which mediate metabolic signaling for development and maintain stress tolerance, likely synergize to support pathogenicity. Oxidative stress resistance protects fungi from host-derived reactive oxygen species during infection [36], while membrane stability modulation may facilitate cell–cell communication during mating or host penetration. The reduced pathogenicity of *SsCYP64* mutants (30% incidence vs. 70% in the wild type) underscores the importance of these combined functions in successful colonization.

## 5. Conclusions

Our findings establish a novel regulatory cascade in *S. scitamineum*: SsCyp64 mediates γ-linolenyl alcohol synthesis to modulate *SsPRF1* transcription, thereby regulating sexual mating/filamentation and pathogenicity. This work highlights the role of cytochrome P450 enzymes as important regulators in of fungal sexual reproduction and provides a theoretical basis for designing targeted strategies to manage sugarcane smut.

## Data Availability

The original contributions presented in this study are included in the article/Supplementary Material. Further inquiries can be directed to the corresponding authors.

## Supplementary Materials

The following supporting information can be downloaded at https://www.mdpi.com/article/10.3390/jof11100729/s1, Figure S1: Phylogenetic and domain architecture analysis of the cytochrome P450 enzyme SsCyp64 in *S. scitamineum*; Figure S2: Generation and molecular verification of the *sscyp64*Δ*-1*, *sscyp64*Δ*-2*, *sscyp64*Δ/*CYP64-1*, and *sscyp64*Δ*/CYP64-2* mutants; Figure S3: RT-qPCR was performed to quantify the transcriptional levels of *SsPRF1*; Figure S4: Exogenous supplementation with 12-hydroxystearic acid, 2-(Methylthiomethyl)furan, and D(+)-Raffinose was performed to assess the sexual mating/filamentation of *SsCYP64* deletion mutants; Table S1: Details of the strains used in this study; Table S2: The primers and sequences used in this study.
