# Supplementary material for: The Cytochrome P450 Enzyme SsCyp64 Mediates γ-linolenyl Alcohol in Regulating Sexual Mating/Filamentation and Pathogenicity of Sporisorium scitamineum
Source: J Fungi (Basel). 2025 Oct 10;11(10):729. doi: 10.3390/jof11100729 (PMC12565241; doi:10.3390/jof11100729)
Supplement: Supplementary file 1 [file jof-11-00729-s001.zip › jof-3880360-supplementary.pdf]

The cytochrome P450 enzyme SsCyp64 mediates γ-linolenyl alcohol in regulating sexual mating/filamentation and pathogenicity of *Sporisorium scitamineum*

Enping Cai,[1,†] Bo Xiong,[1,2,†] Qiuping Ling,[1] Xueting Li,[1] Xinglong Chen,[1] Changqing Chang,[2] Jiayun Wu,[1,*] and Nannan Zhang[1,*]

[1] Guangdong Sugarcane Genetic Improvement Engineering Center, Institute of Nanfan & Seed Industry, Guangdong Academy of Sciences, Guangzhou 510316, China;

[2] Guangdong Provincial Key Laboratory of Microbial Signals and Disease Control, Engineering Research Center of Biological Control, Ministry of Education, College of Plant Protection, South China Agricultural University, Guangzhou 510642, China

* Correspondence author. E-mail address: jiayunng@163.com (J. Wu); zhangnn23@mail2.sysu.edu.cn (N. Zhang).

[†] These authors contributed equally to this work.

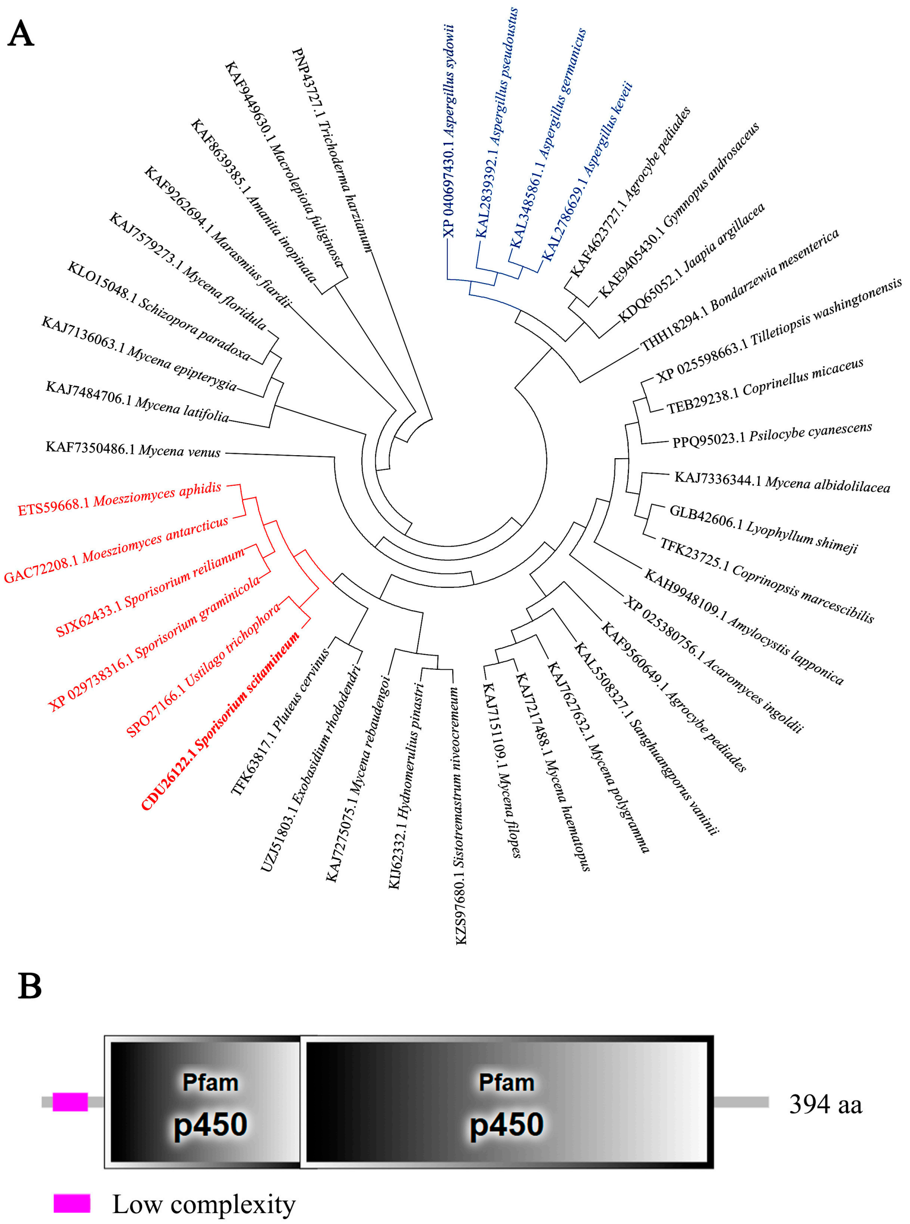

Fig. S1. Phylogenetic and domain architecture analysis of the cytochrome P450 enzyme SsCyp64 in *S. scitamineum*. （A）A Maximum Parsimony phylogenetic tree of Cyp64 proteins was constructed based on sequence data from representative species spanning the ascomycota and basidiomycota. Bootstrap support values along the branches were derived from 1,000 replicate resampling events. Red indicates smut fungi, blue indicates ascomycota, and black indicates basidiomycota. （B）Domain architecture analysis of the SsCyp64 protein, where LCR indicates the presence of a low-complexity region.

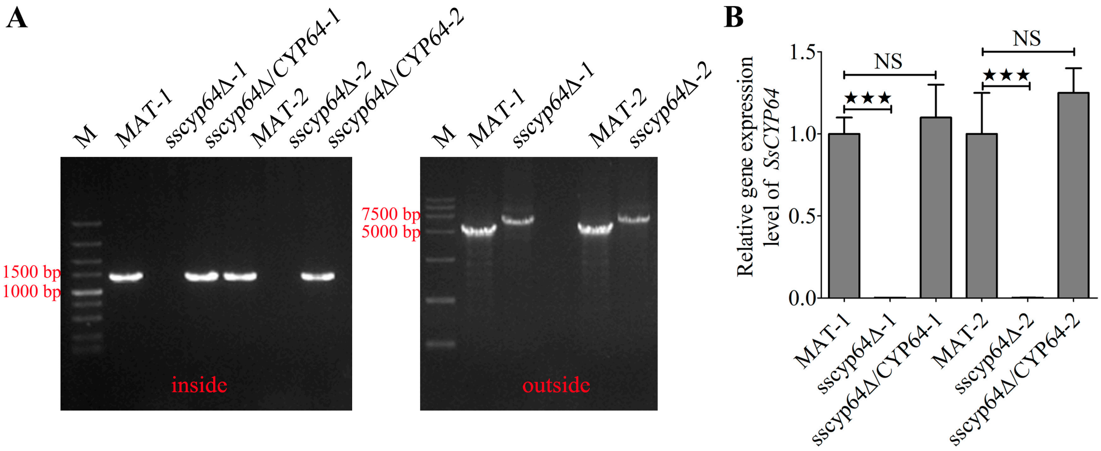

Fig. S2. Generation and molecular verification of the *sscyp64*Δ*-1*, *sscyp64*Δ*-2*, *sscyp64*Δ/*CYP64-1*, and *sscyp6464*Δ/*SsCYP64-2* mutants. （A） PCR amplification was conducted using gene-specific inside-F/R or outside-F/R primers to confirm the replacement of the target gene with the HYG^R selectable marker. Molecular marker sizes (in base pairs, bp) are indicated. （B） RT-qPCR analysis of *SsCYP64* transcriptional expression. Fungal strains were cultured in YePSA medium for 24 hours prior to total RNA extraction. RT-qPCR was subsequently carried out using *ACTIN* as the internal reference gene. The experiment was performed with three biological replicates, each accompanied by three technical replicates. The error bars represent the means ± standard errors of the means (SEMs). Statistical significance was evaluated using one-way analysis of variance (ANOVA) followed by Bonferroni's multiple-comparison test (★★★$P < 0.001$; NS, not significant).

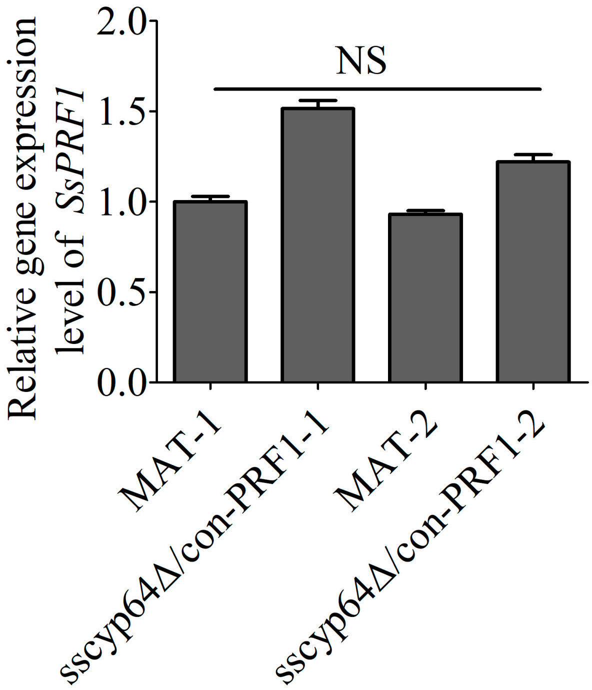

Fig. S3. RT-qPCR was performed to quantify the transcriptional levels of *SsPRF1*. The wild-type (*MAT-1* and *MAT-2*) and *SsPRF1* constitutive expression strains (*ssphacA*Δ/con-*PRF1-1* and *ssphacA*Δ/con-*PRF1-2*) were cultured in YePSA medium for 24 hours, and then total RNA was extracted. The transcriptional level of *SsPRF1* was quantitatively analyzed using RT-qPCR, with *ACTIN* serving as the reference gene. The experiment included three biological replicates, each with three technical replicates. The error bars represent the means ± standard errors of the means (SEMs). Statistical significance of differences was evaluated using one-way analysis of variance (ANOVA) followed by Bonferroni's multiple-comparison test (NS, not significant).

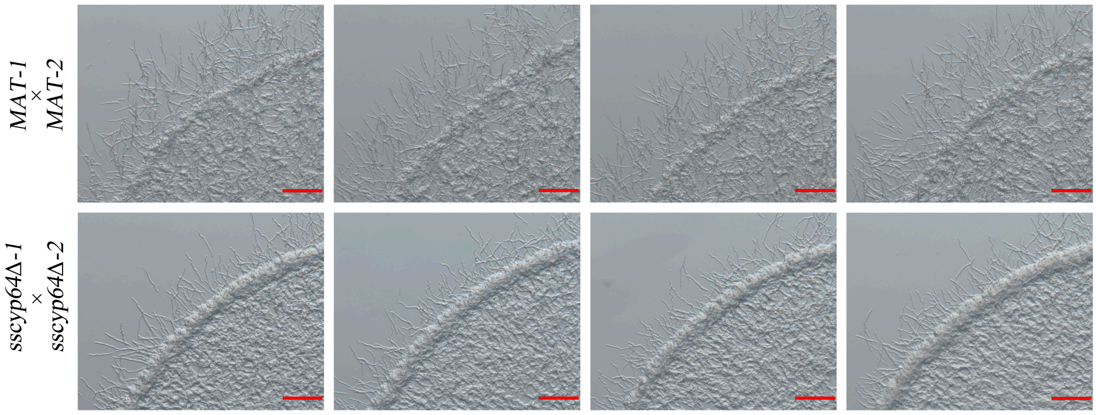

Fig. S4. Exogenous supplementation with 12-hydroxystearic acid, 2-(Methylthiomethyl)furan, and D(+)-Raffinose was performed to assess the sexual mating/filamentation of *SsCYP64* deletion mutants. Haploid cells of distinct genetic mating types were mixed (*MAT-1* × *MAT-2* and *sscyp64*Δ-*1* × *sscyp64*Δ-*2*) in equal volumes and spotted onto minimal medium supplemented with or without 10.0 μM 12-hydroxystearic acid, 2-(Methylthiomethyl)furan, and D(+)-Raffinose. The plates were incubated at 28°C for 18 hours, and the results were photographed. Scale bar is 1.0 mm.

Table

Table S1. Details of the strains used in this study.

Strain	Relevant Genotype	Resistance marker	Source
MAT-1	*a1*, *b1*		Yan et al. 2016
MAT-2	*a2*, *b2*		Yan et al. 2016
sscyp64Δ-1	*a1*, *b1*; *cyp64Δ*	Hygromycin	This study
sscyp64Δ-2	*a2*, *b2*; *cyp64Δ*	Hygromycin	This study
sscyp64Δ/CYP64-1	*a1*, *b1*; *cyp64Δ*; *CYP64*	Zeocin	This study
sscyp64Δ/CYP64-2	*a2*, *b2*; *cyp64Δ*; *CYP64*	Zeocin	This study
sscyp64Δ/con-PRF1-1	*a1*, *b1*; *cyp64Δ*; *con-PRF1*	Zeocin	This study
sscyp64Δ/con-PRF1-2	*a2*, *b2*; *cyp64Δ*; *con-PRF1*	Zeocin	This study

Table S2. The primers and sequences used in this study.

The primers	Sequence (5’- - 3’)
	The primers for genetic deletion
pDAN-F	GTCGTGACTGGGAAAACCCTG
LB-226-R	GGTCAAGACCAATGCGGAGC
pDAN-R	TCACACAGGAAACAGCTATGACC
RB-225-F	GCAAGACCTGCCTGAAACCG
SsCYP64-LB-F	AGGAGAAGAGCGGTGCGATG
SsCYP64-LB-R	GTCGTGACTGGGAAAACCCTGTGAGTTGCGGTTGGGTGTCA
SsCYP64-RB-F	GGTCATAGCTGTTTCCTGTGTGAGGCCAAGAGGTGGATACGCA
SsCYP64-RB-R	GAGCCGTTTGGCGTTACTGC
	The primers for genetic complementation
SsCYP64-com-F	ATCTGATCCAAGCTCAAGCTAGGAGAAGAGCGGTGCGATG
SsCYP64-com-R	AGCAAGATCTAATCAAGCTTAGCAAGCGTCTCAACACCGA
COM-LB-F	CCTCGAGCGATCCTTGAAGC
COM-LB-R	AGCGGGCAGTTCGGTTTCA
COM-RB-F	CAAGAACAAGCGCTGTCGCC

COM-RB-R	CGAGCATTCACTAGGCAACCA
The primers for genetic constitutive expression	
SsPRF1-con-F	AACCAAAACACTCTTCCACCATGCGAGACCAAGCTACCACG
SsPRF1-con-R	AGCAAGATCTAATCAAGCTTCTACGTCGAGGCGGACTGCTG
con-LB-F	GAAAGGTGCGACGGTGTGC
con-LB-R	GGCTGTCGAAAGGTCAGGTCT
con-RB-F	AGTTGACCAGTGCCGTTCCG
con-RB-R	AGCGACGAACCTTGCCATCA
The primers for RT-qPCR	
RT-qPCR-*ACTIN*-F	CAGCTCGATGAAGGTCAAGAT
RT-qPCR-*ACTIN*-R	CACATCTGCTGGAAGGTAGAG
RT-qPCR-*SsCYP86*-F	CGCTCCTTGACCATCAATCT
RT-qPCR-*SsCYP86*-R	GAGTTCGCACTCGACGTAAA
RT-qPCR-*SsPRF1*-F	CAAGCAGTGTCACCGTTAGA
RT-qPCR-*SsPRF1*-R	GGAGAGCAAGGATGCAAGAT
RT-qPCR-*SsMFA1*-F	ATGCTTTCCATCTTTACCCAGA
RT-qPCR-*SsMFA1*-R	GTGCAGCTAGAGTAGCCAAG
RT-qPCR-*SsPRA1*-F	GGACGCTATCACCCAATCTTAC
RT-qPCR-*SsPRA1*-R	TCTCCAACATGGCAACACTC
RT-qPCR-*SsbE*-F	TGAAAGTTCTCATGCAAGCC
RT-qPCR-*SsbE*-R	TGAGAGGTCGATTGAGGTTG
RT-qPCR-*SsbW*-F	CCAGCAGTCCAATGGAGAAA
RT-qPCR-*SsbW*-R	CCAGCAGTCCAATGGAGA
The primers for identification of deletion mutants	
SsCYP64-inside-F	CCACCCGGACCCAAACCTTC
SsCYP64-inside-R	CGTCAAACGGCAACGGATGC
SsCYP86-outside-F	GCTGCTGTCTGGATGCCTGT
SsCYP64-outside-R	GTCTCGGGTGCTCATCCTCG
